# Development and validation of a method for purification of mallein for the diagnosis of glanders in equines

**DOI:** 10.1186/1746-6148-8-154

**Published:** 2012-09-02

**Authors:** Maurício Baltazar de Carvalho Filho, Rodrigo Mauro Ramos, Antônio Augusto Fonseca, Lívia de Lima Orzil, Mariana Lázaro Sales, Vania Lucia de Assis Santana, Marcilia Maria Alves de Souza, Evandro dos Reis Machado, Paulo Rodrigues Lopes Filho, Rômulo Cerqueira Leite, Jenner Karlisson Pimenta dos Reis

**Affiliations:** 1 Laboratório Nacional Agropecuário em Minas Gerais, Pedro Leopoldo, Brazil; 2 Laboratório Nacional Agropecuário em Pernambuco, Recife, Brazil; 3 Instituto Mineiro de Agropecuária, Belo Horizonte, Brazil; 4 Departamento de Medicina Veterinária Preventiva, Escola de Veterinária - Universidade Federal de Minas Gerais, Av. Antonio Carlos 6627, Pampulha, Belo Horizonte, 31270-901, MG, Brazil

**Keywords:** Equine, Glanders, Mallein, Antigen, Diagnosis, Purification

## Abstract

**Background:**

The allergic test of mallein is one of the most frequently used tests, together with the Complement Fixation Test (CFT), for the diagnosis of glanders in endemic areas. Mallein, a purified protein derivative (PPD), is produced similarly to PPD tuberculin and the end product is a primarily proteic antigen, which is only poorly purified. The immuno-allergic activity of mallein is believed to be due to a high molecular weight group of proteins present in the antigen. To improve the quality of the antigen, in terms of sensitivity and specificity, a new method of mallein production was developed, in which purification was accomplished by ultrafiltration in a Tangential Flow Filtration system (TFF).

**Results:**

The TFF methodology efficiently separated the high and low molecular weight protein groups of mallein. The five TFF-purified malleins, produced from *Burkholderia mallei* strains isolated from clinical cases of glanders in Brazil, proved to be more potent than standard mallein in the induction of an allergic reaction in sensitized animals. Regarding specificity, two of the purified malleins were equivalent to the standard and three were less specific.

**Conclusion:**

Some of the TFF-purified malleins showed considerable potential to be used as an auxiliary test in the diagnosis of glanders.

## Background

Glanders occurs primarily in equines, but other species, including humans, may become incidental hosts [1]. The causal agent of glanders is the bacterium *Burkholderia mallei*, a small, Gram negative, non-motile, encapsulated, facultative intracellular rod [2,3]. The disease presents in three main forms: pulmonary, nasal and cutaneous [4]. In horses, glanders is usually chronic, and the infected animal may live for years. In donkeys and mules, the acute form is more common, and the infected animals may die in a few days. Transmission of glanders occurs mainly by the ingestion of food and water contaminated by the secretions of animals with the clinical and sub-clinical forms of the disease and is exacerbated by crowded, unhealthy living conditions. Sharing of grooming and riding equipment is also considered an important form of transmission. [5-7].

Glanders was eradicated from Western Europe and North America [8] but is still present in Asia, the Middle East and South America [9-14]. In Brazil, glanders was first described in 1811 and was most likely introduced by the importation of infected horses from Europe [15]. From 1960 to 1998, the disease was not reported in the country. In 1999, though, it was diagnosed in the Brazilian northeastern states of Pernambuco and Alagoas [14], and subsequently, some foci were reported in other regions of the country [16]. The regulatory legislation for the control and eradication of glanders in Brazil has adopted the CFT and the mallein test for the identification of infected animals. The mallein test is used for confirmation of sub-clinical cases that were positive in the CFT [17]. Both tests have an excellent level of specificity (approximately 100%) [18,19], but the level of sensitivity is suboptimal, with the sensitivity of the CFT being approximately 97% and that of the mallein test being 75.7% [18,20]. Despite the high specificity, false positive results do occur in both tests, which may be related to inadequate purification of the antigen [21]. Verma *et al*. [22] partially purified the maleo-proteins on gel-filtration chromatographic columns, separating fractions of molecular weight above 350 kDa and between 120 and 170 kDa, and verified that the higher molecular weight fraction constituted a better antigen, showing higher sensitivity and specificity in the mallein test than the lower molecular weight fraction.

In this paper we describe a new method for the production and purification of the mallein antigens based on the separation of high and low molecular weight proteins by ultrafiltration in a Tangential Flow Filtration system (TFF). The antigens were purified from mallein pre-concentrates produced from cultures of *B. mallei* strains isolated from clinical cases of glanders in Brazil.

## Results

### Characterization of the B. mallei strains

Two to three days after being inoculated, the guinea pigs developed a bilateral orchitis typical of *B. mallei* infection in this species (Strauss Reaction). The animals were observed for another four to five days and then euthanized, and pure colonies of the bacterium were recovered from purulent material collected from their testicles. Clinical signs and necropsy findings are listed in Table 1. The lack of death or severe illness during the seven-day observation period suggests that the strains are not highly virulent [28].

**Table 1:** **Origen, animal species, year of isolation and virulence of the*B. mallei *strains**

**Strain**	**Animal species**	**Origen**	**Year of isolation**	**Virulence in guinea pigs**		
				**Clinical signs**	**Onset of clinical signs (hours)**	**Organs involved**
1	Mule	Rural area, state of Pernambuco, Brazil	2001	Orchitis	72	Testicles
2	Mule	Rural area, state of Pernambuco, Brazil	2001	Orchitis	48	Testicles and liver
3	Mule	Rural area, state of Alagoas, Brazil	2002	Orchitis	48	Testicles, liver and lungs
4	Horse	City of Recife, state of Pernambuco, Brazil	2002	Orchitis	48	Testicles
5	Horse	City of Recife, state of Pernambuco, Brazil	2002	Orchitis, listlessness	48	Testicles and liver

The five strains of *B. mallei* showed the morphological and staining characteristics of a small, Gram negative, non-spore-forming, irregularly stained rod with bipolar intracellular inclusions. After 48 hours, colonies in 4% glycerol-enriched nutrient agar were small, smooth, cream-colored, moist and viscous, and had the aspect of honey drops when examined against the light.

Table 2 presents the results of the biochemical and molecular tests of the five *B. mallei* strains and the positive control (ATCC 15310). All the other tests of the API 20E kit not shown in the table resulted negative for all the strains. The TSI results indicate that the bacteria do not ferment glucose, sucrose or lactose. The PCR assays did not amplify the *fliP* gene segments of the *B. pseudomallei* strain used as negative control.

**Table 2:** **Results of the biochemical and molecular tests of the*B. mallei *strains and the positive control**

**Tests**	***B. mallei*strains**					
	**1**	**2**	**3**	**4**	**5**	**ATCC 15310**
Catalase	+	+	+	+	+	+
Oxidase	+	+	+	+	+	+
Motility	-	-	-	-	-	-
TSI	RR	RR	RR	RR	RR	RR
L-arginine^*^	+	+	+	+	+	+
Indole^*^	-	-	-	-	-	-
Sodium pyruvate^*^	+	+	+	+	+	+
Gelatinase^*^	+	+	+	+	+	+
Glucose^*^	+	+	+	+	+	+
Sucrose^*^	-	-	-	-	-	-
Inositol^*^	+	+	+	+	+	+
PCR	+	+	+	+	+	+
Real-Time PCR	+	+	+	+	+	+

^*^ Kit API 20E. RR = Butt and slant red.

Although there is considerable disagreement between different authors regarding the biochemical characterization of *B. mallei*, specifically in which sugars are utilized by this bacterium, the results shown here are enough to presumptively classify the samples as *B. mallei*[11,18,29].

### Characterization of the purified malleins

**Figure 1:**
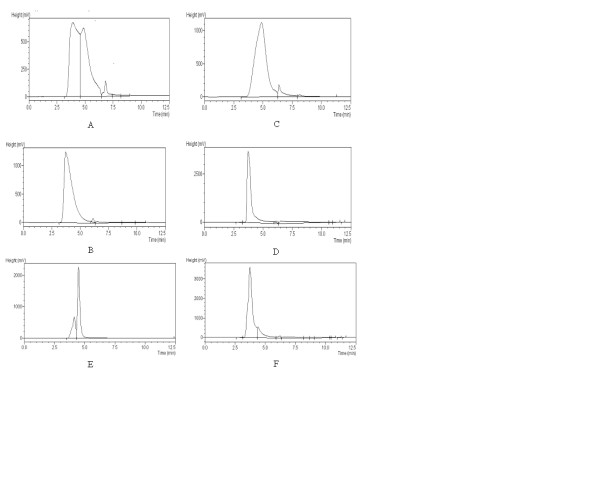
**A: Chromatographic profile of mallein pre-concentrate # 2 showing three distinct peaks at 3.6, 5.0 and 7.0 minutes**; **B**: Chromatographic profile of purified mallein # 2 showing one single peak at 3.6 minutes; **C**: Chromatographic profile of the removed fraction of mallein pre-concentrate # 2 showing two distinct peaks at 5.0 and 6.5 minutes; **D**: Chromatographic profile of bovine thyroglobulin showing one single peak at 3.6 minutes; **E**: Chromatographic profile of bovine albumin showing two distinct peaks at 4.0 and 4.5 minutes.; **F**: Chromatographic profile of purified mallein # 3 showing a well marked peak at 3.6 minutes and a secondary peak at 4.5 minutes.

The resulting protein concentration of the five mallein pre-concentrates was approximately 30 mg/ml, but their chromatographic profiles showed considerable variation, revealing the existence of at least three peaks (Figure 1-A). All of the pre-concentrates, however, showed one peak with a shorter retention time of 3.6 minutes. This peak was our target for purification because all of the other peaks demonstrated retention times indicative of protein fractions of molecular weights below 350 kDa. That way, the purification process was repeated until the chromatographic profile showed a well-marked single peak for a solution with a protein concentration of approximately 10 mg/ml.

After the purification, the retained protein fractions (purified malleins) of four mallein pre-concentrates showed a single peak with a retention time of 3.6 minutes (Figure 1-B), the same retention time of bovine thyroglobulin (Figure 1-D). The exception was purified mallein number 3, which maintained a residual second peak formed at 4.5 minutes (Figure 1-F). On the other hand, the largest peak of the removed protein fractions revealed a retention time between 4.5 and 5.0 minutes (Figure 1-C), similar to the chromatographic profile of bovine albumin (Figure 1-E).

### Potency and specificity tests of the purified malleins

The evaluation of the efficiency of the sensitization of the animals utilized in the potency test by the CFT revealed that they were able to mount an antibody response to the sensitizing emulsion (Table 3).

**Table 3:** **CFT titers for anti-*B. mallei *antibodies in the sera of the sensitized animals utilized in the potency tests**

**Animal**	**CFT titers**						
	**Before 1**^**st**^**sensitization**	**30 days after 1**^**st**^**sensitization**	**30 days after 2**^**nd**^sensitization^*^	**Before 2**^**nd**^**test**	**Before 3**^**rd**^**test**	**Before 4**^**th**^**test**	**Before 5**^**th**^**test**
1	Negative	1:160	1:320	1:40	Negative	Negative	Negative
2	Negative	1:320	1:160	1:80	1:80	1:160	1:80
3	Negative	1:160	1:160	1:160	1:40	1: 80	1:80
4	Negative	1:320	1:160	1:640	1:160	1:320	1:320

^*^Before first test.

The antibody titers rose quickly after the first dose of the sensitizing emulsion and remained stable after the second dose. Subsequently, however, with the exception of animal number 4, the titers dropped slightly, despite the reinforcement doses administered between the tests. Animal number 1, the oldest in the sensitized group, returned to the negative status after the second test but continued to show an allergic response to the malleins.

It should be noted, however, that after the first test, the response to the sensitizing emulsion might have suffered from the influence of the mallein inoculations themselves because it is well known that malleinization may cause seroconversion in healthy animals [19,26,30].

Analysis of variance of the potency and specificity tests revealed no significant difference among the means of the reactions at the four points of inoculation. Only in the case of the potency test of the purified mallein number 3 was there a point x reagent interaction. In this case, there was a significant difference between points 3 and 4 only in the purified mallein. As this difference was not observed in any of the other tests, we chose not to consider it in the comparison of the means.

As is usually the case with biological responses, we expected a high coefficient of variation (CV). Indeed, the CV of the analysis of variance for the potency and specificity tests was high (approximately 30%). For this reason, we used the t-test to compare the means because this test is the most accurate in the presence of high CVs, where the Type II error occurs frequently [31]. Because the points of inoculation did not have a significant effect, we used the general mean effect in the comparisons.

**Figure 2:**
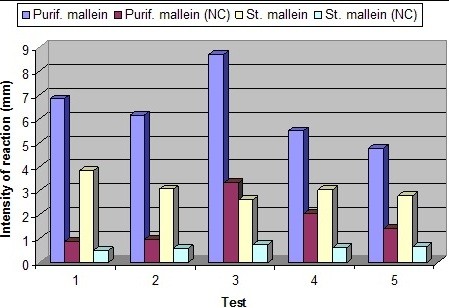
**Results of the potency and specificity tests of the standard and purified malleins.** Purple columns, intensity of the reactions of the potency tests of the five purified malleins (sensitized animals). Red columns, intensity of the reactions of the specificity tests of the five purified malleins (negative controls). Yellow columns, intensity of the reactions of the potency tests of the standard mallein. Green columns, intensity of the reactions of the specificity tests of the standard mallein. Readings in millimeters.

**Figure 3:**
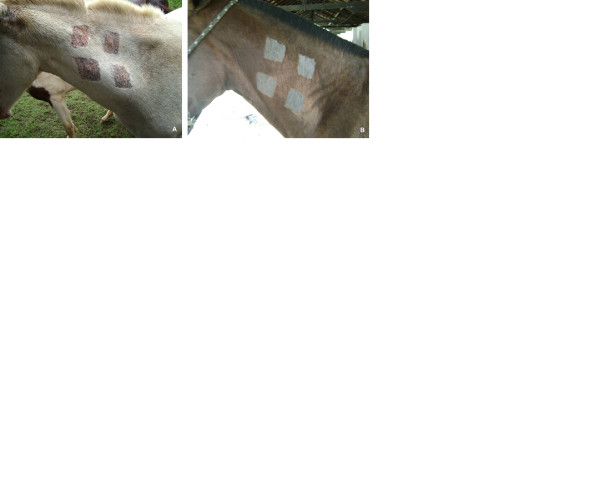
**A: Reactions produced in a sensitized animal by purified mallein number 2 (upper right and lower left points) and by the standard (upper left and lower right points)**; **B**: Results of the inoculation of purified mallein number 2 and the standard in a non-sensitized animal (negative control).

The statistical analysis of the data from the potency tests showed that all of the purified malleins produced reactions significantly larger than those produced by the standard mallein (Table 4 and Figures 2 and 3-A).

**Table 4:** Comparison of the means of the reactions produced by the purified malleins and the standard mallein in the sensitized and control animals

**Reagent**	**Test**									
	**1**		**2**		**3**		**4**		**5**	
	**SA**	**NC**	**SA**	**NC**	**SA**	**NC**	**SA**	**NC**	**SA**	**NC**
Purified malleins	6.86Aa	0.86Ab	6.16Aa	0.96Ab	8.72Aa	3.32Ab	5.52Aa	2.04Ab	4.78Aa	1.42Ab
Standard	3.83Ba	0.48Ab	3.06Ba	0.59Ab	2.63Ba	0.73Bb	3.03Ba	0.61Bb	2.80Ba	0.65Bb

Upper-case letters are used to compare the two reagents (purified mallein and standard) in the same group of animals (sensitized or non-sensitized). Lower-case letters are used to compare the reactions of the same reagent (purified mallein or standard) in the two groups of animals (sensitized and non-sensitized). Means followed by different letters, upper-case in the columns and lower-case in the lines, differ significantly by the t-test (P < 0.01). *SA* Sensitized animals, *NC* Negative controls. Readings in millimeters.

The reactions produced by the purified malleins and by the standard mallein were significantly smaller in the negative controls than those produced in the sensitized animals (Table 4 and Figures 2 and 3-A and B). Of the five purified malleins tested, only the number 3 showed a mean reaction greater than 3 mm in the non-sensitized group. For the standard, the reactions were even smaller, with the strongest reaction again occurring in the negative controls of the third test (0.73 mm). The standard mallein elicited significantly smaller reactions than the purified malleins number 3, 4 and 5 in the control group. For the purified malleins number 1 and 2, however, this difference was not significant (Table 4 and Figure 2).

Among the purified malleins, the number 1 showed the best specific activity, with a potency to specificity ratio of 7.98, and the number 3 showed the worse specific activity, with a potency to specificity ratio of 2.63. The mean potency to specificity ratio for the standard mallein in the five tests was 5.21 (Table 5).

**Table 5:** Specific activity (potency to specificity ratio) of the purified malleins and the standard mallein

**Test**									
**1**		**2**		**3**		**4**		**5**	
PM	SM	PM	SM	PM	SM	PM	SM	PM	SM
7.98	7.98	6.42	5.19	2.63	3.60	2.71	4.97	3.37	4.31

*PM* Purified mallein, *SM* Standard mallein.

### Field test

The results of the field test are included in Table 6. Five out of the 15 animals tested were found positive for both the purified mallein number 2 and the standard, and nine were found negative also for the two reagents. Only in one animal, where purified mallein number 2 was negative and the standard positive, the results did not match. The data, although obtained from a relatively small number of animals, give a Kappa coefficient of 0.86, which is considered an almost perfect agreement (Table 6).

**Table 6:** Agreement between the results of purified mallein number 2 and the standard mallein obtained from 15 suspected cases of glander in horses

**Kappa = 0.86**		**Purified mallein # 2**	
		**+**	**-**
Standard	+	5	0
	-	1	9

## Discussion

The molecular weight of the purified mallein coincided with that of bovine thyroglobulin (660–690 kDa), which agrees with the work of Verma et al. [22] for proteins in this fraction (> 350 kDa). The removed fraction, however, showed a molecular weight similar to that of bovine albumin (67 kDa), which differs from the results of the above cited study, whose authors found a molecular weight of 120–170 kDa for this fraction. To separate the two fractions, however, those authors used a gel-filtration chromatographic column, collecting aliquots of the two main peaks identified in the chromatographic profile of the raw mallein, which allow us to obtain a better purified product, consisting of a more homogeneous group of proteins. Our removed fraction, however, was not purified, but rather concentrated to elevate its protein concentration after the separation of the high molecular weight group of proteins. The resulting product consisted of various sub-fractions, which may have contributed to the increase in the retention time of its main peak following concentration.

The reactions obtained in the potency and specificity tests of this study were considerably smaller than those reported by Verma *et al*. [22] for the majority of the purified malleins they tested in similar experiments. For the standard mallein alone, the mean of the reactions obtained by the cited authors was 30 ± 5 mm in the sensitized animals and 6 ± 3 mm in the negative controls, and some of the purified malleins were equally potent or even more potent than the standard. For the production of the sensitizing emulsion, those authors used two strains of *B. mallei* considered to be highly virulent (Zagreb and Bogor), which may have led to a higher sensitizing capacity. A positive correlation between virulence of the strain and potency of the mallein has already been suggested by Verma *et al*. [22] themselves. The strains of *B. mallei* we used to produce the sensitizing emulsion were only slightly virulent when tested in guinea pigs. If the correlation suggested by the above cited authors does exist, and if it’s in any way reasonable to extend it to the sensitizing capacity of the strain, so the difference in the magnitude of the reactions found in these two studies could be explained by the naturally low reactivity of the malleins produced by the local strains of *B. mallei* and by the low sensitizing capacity of these strains. Furthermore, the efficiency of the sensitizing procedure might have been overestimated by monitoring the antibody responses of the sensitized animals, provided that the allergic reaction elicited by mallein is cell-mediated, as believed.

The standard mallein used in this experiment proved to be poorly reactive. The reactions it produced in the sensitized animals were rather small when compared to the reactions produced by the purified malleins. Its lack of reactivity, however, might be related to antigenic variation between the *B. mallei* strains used in its production and the local ones used to produce the sensitizing emulsion. This possibility demonstrates the necessity of producing diagnostic reagents with antigenic affinity to the agents occurring in the region. However, the reactions produced by the standard mallein in the negative controls were very discrete, suggesting it has a high level of specificity.

Two of the five purified malleins produced in this experiment, malleins number 1 and 2, fared better than the other three in the potency and specificity tests. They proved to be reasonably potent and fairly specific, with potency to specificity ratios of 7.98 and 6.42, respectively. Purified mallein number 2 was further tested in naturally infected animals and found to be equivalent to the standard, regarding its sensitivity and specificity. Purified mallein number 3 was the most potent, but the reactions it produced in the negative controls suggest that it might be non-specific. This purified mallein was the least pure, as demonstrated by HPLC, because we had to interrupt its purification to preserve its protein concentration. However, we cannot rule out the possibility of some cross-reactivity for the mallein antigen in the animals used as negative controls in the specificity test of this purified mallein, because it was in this group of animals that the standard mallein also produced its largest reactions. The other two purified malleins, number 4 and 5, showed intermediate results in the potency and specificity tests and therefore make less ideal candidates for safe antigens in the diagnoses of glanders than the first two.

## Conclusions

The local *B. mallei* strains have demonstrated considerable potential to produce mallein. The TFF methodology was efficient in partially purifying the malleo-proteins, separating fractions of high and low molecular weight. The purified malleins were shown to have molecular weights similar to bovine thyroglobulin (660–690 kDa). The purified malleins produced from local strains of *B. mallei* are more potent and, in three out of five cases, demonstrate less specificity than the standard used in this experiment.

This study demonstrated the efficiency of the TFF methodology in partially purifying soluble proteic antigens, provided additional information about a poorly characterized antigen and revealed the malleinogenic potential of some indigenous strains of *B. mallei* in Brazil.

## Methods

### Origin and characterization of the B. mallei strains

The five strains of *B. mallei* used to produce mallein in this work were isolated from clinical cases of glanders in horses and mules in the Brazilian states of Alagoas and Pernambuco. They were obtained from the Laboratório de Bacteriologia do Setor de Veterinária da Universidade Federal Rural de Pernambuco, Brasil and were designated with the numbers 1 to 5. The origin and characterization of the *B mallei* strains are detailed in Table 1.

Prior to the biochemical and molecular characterization (PCR and Real-Time PCR), the *B. mallei* strains were passaged through guinea pigs in order to select more virulent clones of better maleogenic potential. The guinea pig passage was done by the inoculation of five animals, one for each strain, by the intraperitoneal route with 1.0 ml of a *B. mallei* suspension in PBS containing 10^8^ UFC [23].

The biochemical characterization was performed using the API 20E® kit plus the tests for oxidase, catalase, motility and triple sugar iron (TSI) [24]. The PCR and Real-Time PCR assays were executed according to the OIE Manual of Diagnostic Tests and Vaccines for Terrestrial Animals [25] using oligonucleotides designed on the basis of the differences in the sequences of the *fliP* gene of *B. mallei* and *Burkholderia pseudomallei.* For the Real-Time PCR, we used the RealQ PCR Master Mix® kit from Ampliqon, Odense, Denmark, and primers and probes from IDT DNA® in an Applied Biosystems ABI 7500® thermocycler. The annealing temperature was changed from 63°C to 62°C to adapt the protocol to our Real-Time PCR kit. The new temperature offered the best compromise between specificity and DNA polymerase activity. As a negative control, we used a *B. pseudomallei* strain provided by the Laboratório Central de Saúde Pública do Ceará – LACEN-Ce, and the positive control was a *B. mallei* strain from ATCC (15310).

### Production of the mallein pre-concentrate

The five mallein pre-concentrates were produced according to the method of Huitema [26]. This procedure is very similar to that used to produce PPD tuberculin, where the protein purification is achieved by precipitation with trichloacetic acid (TCA). In brief, cultures of *B. mallei* were grown in Dorset-Henley broth for eight weeks at 37°C and were then steam-inactivated in an autoclave for one hour, tested to confirm the inactivation, centrifuged at 6000 x *g* for one hour, and the supernatant was filtered through a 0.22 μm PVDF membrane to remove the residual cells. This product, called crude mallein, was further processed by the addition of TCA to concentrate the maleo-proteins by precipitation. The precipitate was washed with 5% saline until the pH of the supernatant rose to 2.4, then with acidified saline (pH 3.0) until the pH of the supernatant rose further to 2.7-2.8. It was then dissolved in a minimum quantity of alkaline solution to produce the mallein pre-concentrate, the pH of which must be approximately 6.7. The protein concentration of this mallein pre-concentrate was estimated spectrophotometrically by the Modified Biuret method using a standard curve of albumin with reading at 544 nm [27].

### Purification of the mallein pre-concentrate

The objective of the purification process was to select the proteins with molecular weights above 350 kDa, which are considered to be more immuno-reactive and specific than those of lower molecular weight [22]. The purification of the mallein pre-concentrate was achieved by ultrafiltration in a Millipore Labscale® TFF system fitted with a Pellicon® XL Ultracel 300 device encasing a regenerated cellulose membrane with a molecular weight limit (MWL) of 300 kDa. The mallein pre-concentrate was diluted in 0.01 Mol/L PBS, pH 7.2-7.4, to a total volume of 500 ml, which is the maximum capacity of the TFF equipment reservoir. The diluted pre-concentrate was ultrafiltered until its volume was reduced to approximately 50 ml (one tenth of the initial volume), and the steps of dilution and ultrafiltration were repeated until we achieved the desired purification. The process was monitored by evaluation of the protein content and the chromatographic profile of the product. The protein content was estimated by the modified Biuret method described above, and the chromatographic profile was established by a High Performance Liquid Chromatography (HPLC) system using an Agilent Zorbax® Bio Series GF-250 column with an internal diameter of 4.6 mm. This methodology employs as mobile phase the same PBS solution used to wash the mallein pre-concentrate in the purification process and uses a flow rate of 0.5 ml/min., an injection volume of 50 μL and a UV detector set to 210 nm. The HPLC equipment was a Shimadzu, model LC-20AT. Prior to injection, the sample was diluted with the mobile phase to yield a protein concentration of 1.0 mg/ml.

To protect the proteins during the freeze-drying process, the purified malleins, retentate of the TFF process, were mixed with equal volumes of a buffered 14% glucose solution [26]. This product was filtered through a 0.22 μm PVDF membrane, freeze-dried in 1.0 ml volumes and identified with the same number (1 to 5) as the *B. mallei* strain from which they originated. Prior to use, the purified malleins were rehydrated and diluted in a sterile 0.5% phenol solution to a final protein concentration of 1.0 mg/ml.

The permeate of the TFF process (proteins with molecular weights below 350 kDa) was concentrated using the same TFF equipment fitted with a Pellicon® XL Biomax 5 device encasing a polyethersulfone membrane with MWL of 5 kDa. This concentrate was mixed with equal volumes of the same buffered glucose solution used to dilute the retentate, filtered and freeze-dried.

### Characterization of the purified malleins

The purified malleins were characterized by HPLC using the methodology described above. As a molecular weight reference for the purified fraction (proteins with molecular weights above 350 kDa) we used bovine thyroglobulin (SIGMA), which has a molecular weight between 660 and 690 kDa. As a reference for the removed fraction (proteins with molecular weights below 350 kDa), we used bovine albumin (SIGMA – Fraction V), which has a molecular weight of 67 kDa.

### Potency and specificity tests of the purified malleins

To test the potency of the purified malleins, we used four mixed-breed, non-pregnant mares between 4 and 10 years of age. The animals were sensitized with a 25% packed volume inactivated suspension of *B. mallei* prepared with 3 of the 5 strains used to produce the malleins, according to the method described by Verma *et al*. [22]. The three strains used to produce the suspension were selected on the basis of their virulence as evaluated by inoculation in guinea pigs (Table 1). The cell suspension was diluted in PBS and Freund’s incomplete adjuvant at a ratio of 1:1.5:2.5, respectively, and emulsified. The animals were inoculated with 1.0 ml of the emulsion, with 0.5 ml injected intramuscularly and 0.5 ml injected subcutaneously. A second dose was administered 30 days after the first, and the first potency test was performed 44 days after the second dose. Additional reinforcement doses were administered 30 days after each potency test and the animals were reutilized 30 days after each reinforcement dose to test the next purified mallein. On the whole, the four sensitized animals were used five times, to test the five purified malleins. To evaluate the efficiency of the sensitization, serum samples were collected immediately before the first dose of sensitizing emulsion, 30 days after the first and second doses, and immediately before each test. The sera were tested for the presence of anti-*B.mallei* antibodies by the CFT.

The purified malleins were tested in the same order as their number identification, from 1 to 5. The potency of the purified malleins was evaluated by comparison with a standard mallein (Pasteur Institute, Bucharest, Romania). For each horse out of the four used, four 6 cm^2^ sites were prepared by shaving the skin at each side of the neck, with two sites on an upper line and two on a lower line, vertically and laterally separated from each other by 6 cm. The thickness of the skin fold at each site was measured with a caliper and recorded, and 0.1 ml of mallein, at a concentration of 1.0 mg/ml, was inoculated intradermally at a point in the center of each site in a systematic way such that on the right side of the neck, the purified mallein was inoculated at points 1 and 4, and the standard was inoculated at points 2 and 3, and the order was inverted on the left side of the neck. Forty-eight hours after the inoculation of the malleins, the thickness of the skin at each site was measured again, and the intensity of the reaction was registered as the difference, in millimeters, between the two readings. At each individual test, the two malleins were inoculated in the four sensitized animals and the mean of the reactions (16 individual readings) produced by the purified mallein was compared with the mean of the reactions (16 individual readings) produced by the standard mallein.

To evaluate the specificity of the experimental malleins and the standard, the same protocol was applied at each test to four non-sensitized horses (negative controls), and the means of the reactions produced by each reagent in this group were compared to the means of the reactions produced by the same reagent in the sensitized group.

The data were analyzed statistically by a split plot ANOVA, with the reagents (purified and standard malleins) in the plot and the points of inoculation (1 to 4) in the subplot. For the potency test, we used a randomized blocks experimental design, and for the specificity test, we used a fully randomized experimental design.

The use of animals in this experiment had the approval of the Ethics Committee in Animal Experimentation of the Universidade Federal de Minas Gerais, under the reference number of 167/2011.

### Field test

In an attempt to better understand the activity of the purified antigens in a real scenario with naturally infected animals, purified mallein number 2 was submitted to a field test with the standard mallein involving 15 suspected glanderous horses from an endemic region. The comparison was performed by the simultaneous inoculation of 0.1 mL of the purified mallein and the standard at a protein concentration of 1.0 mg/mL, intradermally, in the right and left lower eye-lid, respectively, of each animal. Reading of the results was done 48 hours after the inoculation and it was considered positive a marked lower eye-lid swelling accompanied or not by purulent secretion and conjunctivitis.

## Abbreviations

ANOVA: Analysis of variance; ATCC: American type culture collection; CFT: Complement fixation test; CV: Coefficient of variation; HPLC: High performance liquid chromatography; LACEN-Ce: Laboratório Central de Saúde Pública do Ceará; OIE: Office International des Epizooties (World Organization for Animal Health); PBS: Phosphate buffered saline; PCR: Polymerase chain reaction; PPD: Purified protein derivative; PVDF: Polyvinylidene difluoride; TCA: Trichloacetic acid; TFF: Tangential flow filtration; TSI: Triple sugar iron.

## Competing interests

The authors declare that they have no competing interests.

## Authors’ contributions

MBCF: designed the study, interpreted the data and wrote the manuscript; AAFJ and MLS: carried out the PCR and qPCR of the *B. mallei strains*; RMR and LLO: carried out the HPLC essays; ERM and PRLF: carried out the potency and specificity tests; MMAS and VLAS: carried out the CFT tests; RCL: critically revised the manuscript; JKPR: participated in design and coordination of the study and, critically revised the manuscript and gave final approval of the version to be published.
